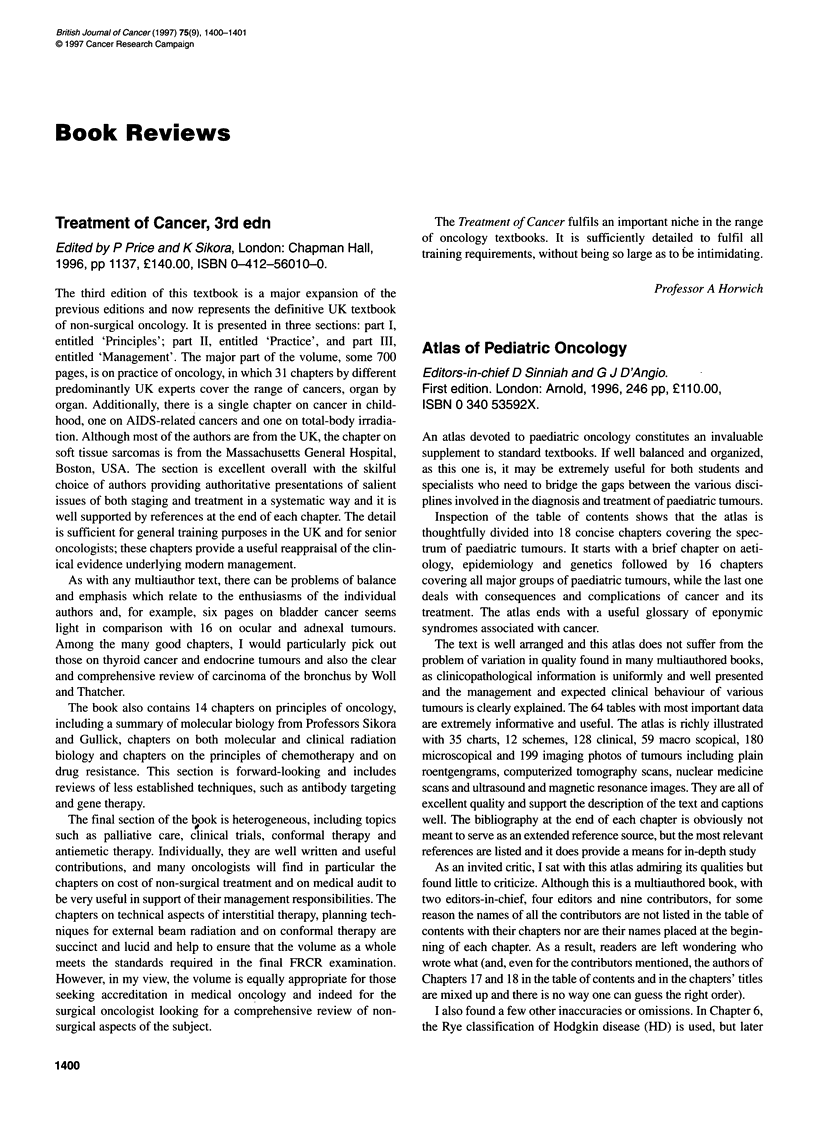# Treatment of Cancer, 3rd edn

**Published:** 1997

**Authors:** A Horwich


British Journal of Cancer (1997) 75(9), 1400–1401
© 1997 Cancer Research Campaign

### Book Reviews

### Treatment of Cancer, 3rd edn

*Edited by P Price and K Sikora*, London: Chapman Hall, 1996, pp 1137, £140.00, ISBN 0–412–56010–0.

The third edition of this textbook is a major expansion of the previous editions and now represents the definitive UK textbook of non-surgical oncology. It is presented in three sections: part I, entitled 'Principles'; part II, entitled 'Practice', and part III, entitled 'Management'. The major part of the volume, some 700 pages, is on practice of oncology, in which 31 chapters by different predominantly UK experts cover the range of cancers, organ by organ. Additionally, there is a single chapter on cancer in childhood, one on AIDS-related cancers and one on total-body irradiation. Although most of the authors are from the UK, the chapter on soft tissue sarcomas is from the Massachusetts General Hospital, Boston, USA. The section is excellent overall with the skilful choice of authors providing authoritative presentations of salient issues of both staging and treatment in a systematic way and it is well supported by references at the end of each chapter. The detail is sufficient for general training purposes in the UK and for senior oncologists; these chapters provide a useful reappraisal of the clinical evidence underlying modern management.

As with any multiauthor text, there can be problems of balance and emphasis which relate to the enthusiasms of the individual authors and, for example, six pages on bladder cancer seems light in comparison with 16 on ocular and adnexal tumours. Among the many good chapters, I would particularly pick out those on thyroid cancer and endocrine tumours and also the clear and comprehensive review of carcinoma of the bronchus by Woll and Thatcher.

The book also contains 14 chapters on principles of oncology, including a summary of molecular biology from Professors Sikora and Gullick, chapters on both molecular and clinical radiation biology and chapters on the principles of chemotherapy and on drug resistance. This section is forward-looking and includes reviews of less established techniques, such as antibody targeting and gene therapy.

The final section of the book is heterogeneous, including topics such as palliative care, clinical trials, conformal therapy and antiemetic therapy. Individually, they are well written and useful contributions, and many oncologists will find in particular the chapters on cost of non-surgical treatment and on medical audit to be very useful in support of their management responsibilities. The chapters on technical aspects of interstitial therapy, planning techniques for external beam radiation and on conformal therapy are succinct and lucid and help to ensure that the volume as a whole meets the standards required in the final FRCR examination. However, in my view, the volume is equally appropriate for those seeking accreditation in medical oncology and indeed for the surgical oncologist looking for a comprehensive review of non-surgical aspects of the subject.

The *Treatment of Cancer* fulfils an important niche in the range of oncology textbooks. It is sufficiently detailed to fulfil all training requirements, without being so large as to be intimidating.

*Professor A Horwich*